# Prostacyclin Affects the Relation Between Brain Interstitial Glycerol and Cerebrovascular Pressure Reactivity in Severe Traumatic Brain Injury

**DOI:** 10.1007/s12028-019-00741-4

**Published:** 2019-05-23

**Authors:** Lars-Owe D. Koskinen, Nina Sundström, Linda Hägglund, Anders Eklund, Magnus Olivecrona

**Affiliations:** 1 Department of Pharmacology and Clinical Neuroscience, Neurosurgery, Umeå University, 901 85 Umeå, Sweden; 2 Department of Radiation Sciences, Biomedical Engineering, Umeå University, Umeå, Sweden; 3 Department of Anaesthesia and Intensive Care, Section for Neurosurgery, Faculty of Health and Medicine, Department for Medical Sciences, Örebro University, Örebro, Sweden

**Keywords:** Traumatic brain injury, Pressure reactivity, Autoregulation, Glycerol, Cerebral microdialysis, Prostacyclin

## Abstract

**Background:**

Cerebral injury may alter the autoregulation of cerebral blood flow. One index for describing cerebrovascular state is the pressure reactivity (PR). Little is known of whether PR is associated with measures of brain metabolism and indicators of ischemia and cell damage. The aim of this investigation was to explore whether increased interstitial levels of glycerol, a marker of cell membrane damage, are associated with PR, and if prostacyclin, a membrane stabilizer and regulator of the microcirculation, may affect this association in a beneficial way.

**Materials and Methods:**

Patients suffering severe traumatic brain injury (sTBI) were treated according to an intracranial pressure (ICP)-targeted therapy based on the Lund concept and randomized to an add-on treatment with prostacyclin or placebo. Inclusion criteria were verified blunt head trauma, Glasgow Coma Score ≤ 8, age 15–70 years, and a first measured cerebral perfusion pressure of ≥ 10 mmHg. Multimodal monitoring was applied. A brain microdialysis catheter was placed on the worst affected side, close to the penumbra zone. Mean (glycerol_mean_) and maximal glycerol (glycerol_max_) during the 96-h sampling period were calculated. The mean PR was calculated as the ICP/mean arterial pressure (MAP) regression coefficient based on hourly mean ICP and MAP during the first 96 h.

**Results:**

Of the 48 included patients, 45 had valid glycerol and PR measurements available. PR was higher in the placebo group as compared to the prostacyclin group (*p* = 0.0164). There was a positive correlation between PR and the glycerol_mean_ (*ρ* = 0.503, *p* = 0.01) and glycerol_max_ (*ρ* = 0.490, *p* = 0.015) levels in the placebo group only.

**Conclusions:**

PR is correlated to the glycerol level in patients suffering from sTBI, a relationship that is not seen in the group treated with prostacyclin. Glycerol has been associated with membrane degradation and may support glycerol as a biomarker for vascular endothelial breakdown. Such a breakdown may impair the regulation of cerebrovascular PR.

## Introduction

Cerebral injury may affect the autoregulation of cerebral blood flow (CBF_AR_). Thus, it is important in traumatic brain injury (TBI) patients to understand if the CBF_AR_ is intact. This may help in optimizing the treatment in order to secure an adequate cerebral blood flow. As direct measurements of the autoregulatory state of the cerebral blood flow are difficult, surrogate methods have been introduced, including, among others, pressure reactivity indices such as PR [1, 2] and PR_x_ [3], being applied as research tools.

A central function of cerebral pressure autoregulation is the ability to maintain a stable intracranial pressure (ICP) for a wide range of mean arterial blood pressure (MAP) levels. We implemented the PR-method by Howell et al. [1]. They assessed the patient’s ability to maintain a stable ICP for a wide range of MAP levels by calculating the correlation between hourly mean ICP and MAP values. Patients were described as pressure passive if ICP varied with MAP and pressure active if ICP varied inversely with MAP. If ICP was stable over a wide range of MAP levels, then the patient was described as pressure stable. The slope of the regression line was defined as a measure of pressure reactivity PR. PR has been shown to correlate with outcome and to be dependent on treatment regime [1]. It is important to understand that PR is not equivalent with PR_x_ that uses a much shorter time domain and is calculated as a moving correlation coefficient [3, 4]. However, none of the methods have been compared to a gold-standard method measuring cerebral blood flow continuously over a wide range of arterial blood pressure including the lower and upper limit of CBF_AR_ in the human being.

From a clinical point of view, a solid and easy-to-use surrogate measure of CBF_AR_ is of utmost importance and may help in individualizing the neuro-intensive treatment. Little is known of whether these cerebral autoregulatory measurements correlate to other parameters mirroring the metabolic state of the injured brain [5]. In a situation with cell membrane degradation due to injury, a related vascular endothelium dysfunction might occur. This could further affect the cerebral autoregulation mediated by the endothelium.

Prostacyclin has been shown to possess a variety of biological functions including a vasodilating and membrane stabilizing effect [6–9] to inhibit leukocyte adhesion, platelet aggregation and to improve microcirculation [8, 10]. It has previously been reported that prostacyclin affects the pressure reactivity [11] and the inflammatory response after TBI [12]. A possible effect on cerebral vasospasm after subarachnoid hemorrhage (SAH) has been reported [13].

Our hypothesis was that prostacyclin has beneficial effects on cell membrane damage which are mirrored in extracellular glycerol and that this is associated with vascular regulation measured as PR. We have previously reported an effect of prostacyclin on PR [11] and have now extended the study to explore the association of cerebral glycerol in relation to PR [1, 2] and prostacyclin treatment.

## Materials and Methods

### Patients

The patient cohort was investigated in a prospective consecutive double-blinded randomized study on the effect of prostacyclin as an add-on therapy in patients with severe TBI (sTBI) [14]. In short, the inclusion criteria were verified non-penetrating sTBI, Glasgow Coma Score (GCS) at intubation and sedation ≤ 8, age 15–70 years, a first measured cerebral perfusion pressure (CPP) of 10 mmHg or more, and arrival in our department within 24 h of trauma. This strict approach was chosen in order not to introduce a bias such as “judged not to survive,” “not expected to benefit from treatment,” etc. Exclusion criteria were pregnant or breast-feeding woman, known bleeding disorder, known allergy to epoprostenol, and penetrating head injury. The study accepted subjects with GCS 3 and dilated fixed pupils as long as the initial CPP was 10 mmHg or above.

### General Monitoring

An intra-parenchymal ICP measuring device (Codman MicroSensor™, Johnson & Johnson Professional Inc., Raynham, MA, USA) was implanted as soon as possible. This ICP sensor has been shown to be reliable with very low drift and low complication rate [15, 16]. If a ventriculostomy was applied, it was mainly used to drain cerebrospinal fluid for treating ICP elevations. The drain was kept closed except for drainage of minimal amounts of fluid as needed.

Invasive arterial blood pressure was continuously monitored and the reference level set at the heart level. MAP and CPP were calculated using the monitoring equipment (Marquette Solar, General Electric Medical Systems, Milwaukee, Wisconsin, USA). No correction of the CPP was indicated as all patients were treated in a position without head elevation.

### Microdialysis

Two microdialysis catheters with gold tip (CMA 70, CMA Microdialysis AB, Solna, Sweden) were placed in the brain parenchyma in a standardized fashion frontally on each side approximately at the Kocher’s point. In this study, we used the results from the side that initially, by the clinician, was designated to be the most injured. The microdialysis catheter tip distance from the lesion, such as hematoma, evacuated hematoma, or contusion, was calculated using the computed tomography (CT) scans. In case of diffuse injury, the catheter tip was in the edematous tissue and these subjects were not included in the calculated distances. CMA 106 or 107 microdialysis pumps (CMA Microdialysis AB) were used. The perfusion flow rate was 0.3 µl/min, and the “Perfusion fluid CNS” (CMA Microdialysis AB) was used. The first dialysate was discarded, and the sampling of the first analyzed sample was started 0.5–2.5 h after the start of the microdialysis. The sampling time for each sample was 2 h. If not directly analyzed, the filled vials were stored in a refrigerator for no more than 24 h and then frozen to − 70 °C. The samples were analyzed as soon as possible with a CMA 600 analyzer (CMA Microdialysis AB). In this report, the glycerol levels are reported and the results categorized as the mean of the first 96 h (glycerol_mean_) and the maximal (glycerol_max_) level during the sampling period. The glycerol_max_ value may reflect the severity of the tissue damage better than the mean. The glycerol_mean_ of the first 96 h of sampling was chosen to represent the worst clinical scenario as, after this period, the glycerol often starts to decline. This equals the period of prostacyclin infusion. The lactate/pyruvate ratio during the same period was calculated in order to control for a metabolic difference between the groups.

### Pressure Reactivity Index

PR has been considered as a measure of cerebrovascular autoregulation [2]. Based on minute values, the hourly mean MAP and ICP were calculated. Individual hourly mean values were plotted as ICP versus MAP for the first 96 h of treatment and the regression line calculated. The regression coefficient for each subject represents the PR [1, 11].

### Data Handling and CT Classification

Physiological parameters from the intensive care unit (ICU) system (Marquette Solar, General Electric Medical Systems) were stored on a computer using the LABpilot software (CMA Microdialysis AB). The data were simultaneously stored in the patient’s case file in the ICU system (Picis, Inc, Wakefield, MA, USA). The microdialysis data were transferred from the CMA 600 analyzer to a personal computer and further processed with the LABpilot software. Every case file was scrutinized for outliers due to, for example, calibration periods and mechanical disturbances. Furthermore, the correlation in time of the sampling values was controlled.

The brain tissue injury was evaluated by using the first CT scan of the head characterized according to the Rotterdam scoring system [17]. The scoring was repeated using a new scan at 24 h after trauma.

### Treatment

An ICP-targeted treatment protocol based on the Lund concept was used [18, 19]. In short, all subjects were continuously sedated, using midazolam and fentanyl, and artificially ventilated (PaO_2_ > 12 kPa, PaCO_2_ 4.5–5.5 kPa). Normovolemia and normal colloid osmotic pressure were maintained using infusion of packed red blood cells, albumin, Ringer’s acetate, and glucose solutions, with the goal being a neutral fluid balance. When normovolemia and a controlled cardiovascular situation were achieved, clonidine and metoprolol were administered in order to reduce the capillary hydrostatic pressure and the general level of stress in the subjects. If needed in order to control the ICP, a low dose of thiopental infusion was administered. Further interventions included ventriculostomy for drainage of CSF, and uni- or bilateral hemicraniectomy. The subjects were all treated in the supine position without head elevation.

The subjects were randomized to receive prostacyclin (Flolan^®^, GlaxoSmithKline, Brentford, UK) or saline (placebo) [14]. Flolan was administered at a rate of 0.5 ng/kg/min during 72 h and then tapered during 24 h.

### Statistics

The statistical analysis was performed using the JMP (version 11.0.0, SAS Institute Inc. USA) software package. Results are reported as mean ± standard error of the mean (SEM) for continuous variables and in cases of discrete variables as median and range. When applicable, two-sided un-paired *t* test or Wilcoxon rank-sum test were used for comparison of group values. Proportions were evaluated by *χ*^2^-test and Spearman’s rho for correlation analysis. A *p* ≤ 0.05 was considered statistically significant.

## Ethics and Approval

The study was approved by the regional ethics committee (00–175, 05–007 M) and by Läkemedelsverket (Swedish Medical Products Agency) (151:633/01) and performed in accordance with the ethical standards as stated in the declarations of Helsinki. The study is registered as a clinical trial (ClinicalTrial.gov identifier NCT01363583).

## Results

Forty-eight patients (17 female, 31 male) were included in the initial study. In 45 of these, valid glycerol and PR measurements were available. Twenty-one subjects received prostacyclin and twenty-four placebo. All results are based on these 45 subjects (15 females, 30 males). Mean age was 35.5 ± 2.2 years, GCS 6 (3–8), and injury severity score (ISS) 29 (9–50), for more details and demographics, see Table 1. There was no significant difference between the placebo and prostacyclin groups concerning MAP, ICP, CPP, GCS, ISS, time to first CT, Rotterdam score, proportion or timing of hemicraniectomy, Glasgow Outcome Scale Extended, and mortality at 6 months or lactate/pyruvate ratio during the 96 h, see Table 1.

**Table 1:** Demographics and some other parameters in the different groups

	Placebo (*n* = 24)	Prostacyclin (*n* = 21)	*p*
Women (*n*)	6	9	0.2049, *χ*^2^
Men (*n*)	18	12	
Age (years, mean ± SEM)	34.0 ± 2.4	37.1 ± 3.8	0.4846, *t* test
GCS (median, min–max)	6 (3–8)	5 (3–8)	0.2220, Wilcoxon rank-sum
ISS (mean ± SEM)	27.7 ± 2.1	29.7 ± 2.1	0.5230, *t* test
MAP (mmHg, mean ± SEM)	80.1 ± 1.5	82.2 ± 1.1	0.3689, *t* test
ICP (mmHg, mean ± SEM)	18.5 ± 2.6	16.3 ± 0.9	0.4341, *t* test
CPP (mmHg, mean ± SEM)	62.1 ± 2.5	63.9 ± 3.0	0.6479, *t* test
CT scan time from injury (h ± sem)	3.2 ± 0.7	2.8 ± 0.6	0.7257, *t* test
Rotterdam score (median, min–max)			
Initial	3 (1–5)	3 (2–4)	0.9519, Wilcoxon rank-sum
At 24 h after trauma	2.4 (1–5)	3 (2–4)	0.6062
Hemicraniectomy (*n*)	9/24	8/21	0.9672, *χ*^2^
MDL (mm, mean ± SEM)	13.8 ± 2.6	16.3 ± 3.2	0.5559, *t* test
MDD (*n*)	4/24	4/21	0.8349, *χ*^2^
Lactate/pyruvate ratio	46.6 ± 7.4	43.5 ± 4.3	0.8556, Wilcoxon rank-sum
GOSE (median)	4.5 (1–8)	5 (1–8)	0.7895, Wilcoxon rank-sum
Mortality (%)	16.7	14.3	0.8257, *χ*^2^

The statistical comparisons are between the two groups

*CPP* cerebral perfusion pressure, *CT* computed tomography, *GCS* Glasgow coma score, *GOSE* Glasgow outcome scale extended, *ICP* intracranial pressure, *ISS* injury severity score, *MAP* mean arterial blood pressure, *MDD* microdialysis in diffuse injury, *MDL* microdialysis probe distance to lesion

The microdialysis catheter tip was placed at a distance of 15.0 ± 2.0 mm from the lesion and thus in the penumbra zone. The distance or the number of diffuse cases was not different between the placebo and prostacyclin group, see Table 1. The PR and glycerol values separated into placebo and prostacyclin treatments are given in Table 2. The glycerol level tended to be higher in the placebo group compared to the prostacyclin group, but this did not reach statistical significance. The PR was higher in the placebo group compared to the prostacyclin group (*p* = 0.0164).

**Table 2:** PR and glycerol levels in the different groups

	Placebo (*n* = 24)	Prostacyclin (*n* = 21)	*p* * Wilcoxon rank-sum
PR	0.121 ± 0.034	0.024 ± 0.037	0.0164
Glycerol_mean_ (µmol/l)	143.5 ± 28.3	99.0 ± 30.2	0.8112
Glycerol_max_ (µmol/l)	415.2 ± 122.4	317.6 ± 60.6	0.9185

*Placebo versus prostacyclin. Values are mean ± SEM

*PR* pressure reactivity

**Fig. 1:**
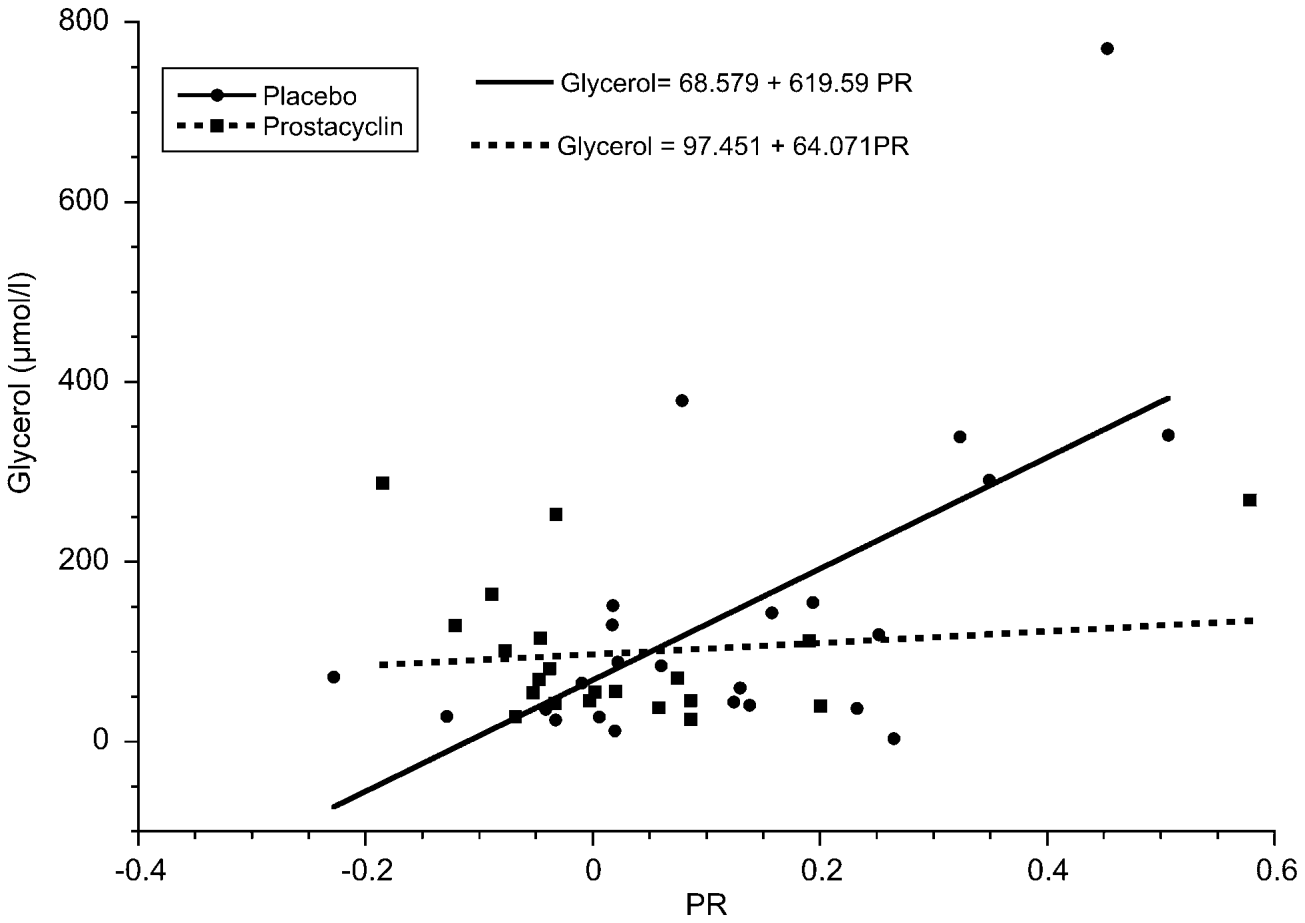
The association between glycerol_mean_ and PR levels in the placebo (*n* = 24, *ρ* = 0.503, *p* = 0.012, Spearman’s Rho) and prostacyclin groups (*n* = 21, *ρ* = − 0.323, *p* = 0.153, Spearman’s Rho). The lines are the linear fit in the two groups

There was a significant positive correlation between glycerol_mean_ (*ρ* = 0.503, *p* = 0.012), glycerol_max_ (*ρ* = 0.490, *p* = 0.015), and PR only in the placebo group, see Table 3 and Fig. 1.

**Table 3:** Correlations of PR in relation to glycerol levels in the different groups

	*ρ*	*p*
Glycerol_mean_		
Placebo (*n* = 24)	0.503	0.012
Prostacyclin (*n* = 21)	− 0.323	0.153
Glycerol_max_		
Placebo (*n* = 24)	0.490	0.015
Prostacyclin (*n* = 21)	− 0.105	0.650

Spearman’s rho correlation

As shown in Fig. 1, there seems to be one outlier in the placebo group. This value is from a patient who was left unattended for 16 h at the scene of trauma. The patient was GCS 4 and hypothermic on arrival at the emergency room. A post hoc analysis excluding this patient showed that the results were still valid.

## Discussion

Our main findings were that prostacyclin affects the PR correlation to cerebral interstitial levels of glycerol and that cerebral interstitial glycerol was correlated to PR suggested to be a biomarker for CBF_AR_.

Complex mechanisms are involved in the regulation of cerebral blood flow [20–22]. The CBF_AR_ [23] is primarily driven by the smooth muscle response to the transmural pressure in the vessel—the Bayliss effect [24, 25] and metabolic and neurohumoral mechanisms [26]. In this, complex mechanisms involving endothelial-dependent mechanisms are involved and it is well known that prostaglandins and arachidonic acid derivates are involved in the regulation of cerebral blood flow and metabolism [27]. It is not surprising that different traumas to the brain may disturb the mechanisms involved in the autoregulation and that metabolic disturbances including cell membrane damage contribute to this [28–31]. Furthermore, various pressure reactivity indices assumed to reflect the CBF_AR_ have been demonstrated to be affected after a variety of cerebral injury [32].

It has been suggested that trauma disturbs the balance between thromboxane and prostacyclin toward thromboxane. This might have negative effects as the biological effects of prostacyclin, including vasodilating and membrane stabilizing effects [6–9], inhibition of leukocyte adhesion and platelet aggregation, and improvement of microcirculation, are counteracted [8, 10]. In clinical studies, prostacyclin has been shown to attenuate the inflammatory response after TBI [12] and to counteract cerebral vasospasm after subarachnoid hemorrhage [13]. This effect was not statistically evident in another cohort of SAH treated in accordance with another regime with the addition of prostacyclin [33]. The authors pointed out several reasons why this may be the case, and advocated further exploration of prostacyclin treatment in SAH.

The intention with the prostacyclin treatment was to counteract the proposed imbalance between thromboxane and prostacyclin. The above-mentioned effects of prostacyclin may normalize the cerebrovascular reactivity and thus the CBF_AR_. It may also affect the interstitial glycerol levels through the breakdown of biological membranes. It is therefore interesting that the correlation between PR and glycerol is attenuated/abolished in the prostacyclin group. One may suggest that, since prostacyclin tends to decrease the interstitial glycerol levels and stops them from being significantly correlated to the PR, it indicates that prostacyclin exerts a biologically beneficial effect on the pathophysiological processes affecting cellular damage and CBF_AR_. However, one may not exclude the possibility that the disturbed pressure reactivity per se is associated with more structural damages rendering opposite effects as suggested for prostacyclin. The present investigation does not solve this question.

As the brain does not contain triglycerides, cerebral interstitial glycerol has been proposed to be a marker of brain cell membrane degradation and thus cell damage due to the degradation of membranous glycerophospholipids [34, 35]. Thus, increasing levels of interstitial glycerol could be a marker, not only for neuron and glial damage but also for endothelial disruption and dysfunction. A consequence of this could be a disturbed CBF_AR_. Our results suggest that glycerol may be an extracellular marker associated with CBF_AR_. The exact pathophysiological meaning of these findings is obscure and to be further explored.

Our results are in line with results reporting a significantly higher glycerol level in perilesional tissue and deranged PR_x_, but an actual correlation could not be confirmed [36]. Other measures of metabolic events and various vascular pressure indices have been studied. L-PRx was not correlated to lactate/pyruvate ratio (LPR), pyruvate, lactate, glutamate or glucose levels in TBI [37]. In TBI patients a daily decrease, although not statistically proven, in PR_x_ concomitant with an increase in extracellular glucose and decrease in LPR indicated an association of the CBF_AR_ and cerebral metabolism [38]. Furthermore, in an experimental animal model of intracerebral hematoma there was no correlation between predefined levels of flow-related or oxygen-related autoregulation indexes and the extracellular cerebral metabolites glucose, lactate, pyruvate, and glutamate [39].

Indeed, there is a pitfall in comparing a global measurement of cerebral dynamics such as PR or PR_x_ with microdialysis mirroring a very local tissue metabolic milieu. Moreover, the autoregulatory state may vary between vascular territories. Reported results in the literature are difficult to compare as the sampling location of the extracellular metabolites varies between studies and is not always described. In our study, the high levels of glycerol and lactate/pyruvate ratio probably mirror the fact that the microdialysis probes were close to the injury. Another pitfall is the highly dynamic evolution of the tissue damage [40] which may result in interpretation difficulties in relation to the microdialysis samples. In the present study, there was no difference in Rotterdam score, thus indicating balanced groups. In clinical practice, hemicraniectomy is a standard procedure in cases with refractory high ICP. Such a surgical removal of skull bone may interfere with the PR measurement. However, in the present investigation, the ratio of hemicraniectomy was equal in the placebo and prostacyclin group. This probably rules out an effect of the procedure on the present results. To further compare the equality between the groups, an eventual difference in LPR was calculated and there was no difference.

As PR is based on hourly mean values, rapid vascular responses in intracranial arterial volume variations are filtered away and thereby also the fluctuations in ICP related to these volume variations. Thus, the slope of ICP vs. MAP as calculated in PR does not describe an autoregulatory response in the same time domain as described by PRx [3]. PR is based on a long-time frame and might still reflect the general condition of the autoregulatory system [1]. In a recent publication, reporting results from the CENTER-TBI study, it states that treatment heterogeneity, time of data collection, and dichotomization of outcome may interfere with the measurements of pressure reactivity indices [41] making it difficult to know how to compare and interpret results from different studies and even more so in respect to different measurements of CBF_AR_. We recognize that PR_x_ is a more dynamic measurement of vascular reactivity than PR and would be the method of choice today. Our prospective consecutive double-blinded randomized study was conducted 10 years ago, and at that time we lacked the suitable equipment for PR_x_ measurements. However, in almost all publications the mean of PR_x_ over several days is reported, thus smoothing the dynamic property of the measurement.

## Conclusion

In summary, PR seems to be correlated to the cerebral interstitial glycerol level, which has been suggested to be a measure of membrane degradation in the brain. This association is not observed in the prostacyclin group. One may speculate that interstitial cerebral glycerol levels, in fact, mirror vascular integrity. We suggest that, by individualizing the treatment of the injured patient, in order to normalize the interstitial cerebral glycerol levels, the vascular pressure reactivity may improve. This might protect the brain from abnormal fluctuations in cerebral blood flow and thus counteract ischemic and/or edema propagation.

## Clinical Trial Registration

The initial study is registered as a clinical trial (ClinicalTrial.gov, identifier NCT01363583).
